# Differentiation of oligodendrocyte progenitor cells from dissociated monolayer and feeder-free cultured pluripotent stem cells

**DOI:** 10.1371/journal.pone.0171947

**Published:** 2017-02-13

**Authors:** Tomoko Yamashita, Yuki Miyamoto, Yoshio Bando, Takashi Ono, Sakurako Kobayashi, Ayano Doi, Toshihiro Araki, Yosuke Kato, Takayuki Shirakawa, Yutaka Suzuki, Junji Yamauchi, Shigetaka Yoshida, Naoya Sato

**Affiliations:** 1 Discovery Research Laboratories, Research Division, Mitsubishi Tanabe Pharma Corporation, Yokohama, Kanagawa, Japan; 2 Department of Pharmacology, National Research Institute for Child Health and Development, Setagaya, Tokyo, Japan; 3 Department of Functional Anatomy and Neuroscience, Asahikawa Medical University, Asahikawa, Hokkaido, Japan; 4 MP Healthcare Venture Management, Boston, Massachusetts, United States of America; Osaka University, JAPAN

## Abstract

Oligodendrocytes myelinate axons and form myelin sheaths in the central nervous system. The development of therapies for demyelinating diseases, including multiple sclerosis and leukodystrophies, is a challenge because the pathogenic mechanisms of disease remain poorly understood. Primate pluripotent stem cell-derived oligodendrocytes are expected to help elucidate the molecular pathogenesis of these diseases. Oligodendrocytes have been successfully differentiated from human pluripotent stem cells. However, it is challenging to prepare large amounts of oligodendrocytes over a short amount of time because of manipulation difficulties under conventional primate pluripotent stem cell culture methods. We developed a proprietary dissociated monolayer and feeder-free culture system to handle pluripotent stem cell cultures. Because the dissociated monolayer and feeder-free culture system improves the quality and growth of primate pluripotent stem cells, these cells could potentially be differentiated into any desired functional cells and consistently cultured in large-scale conditions. In the current study, oligodendrocyte progenitor cells and mature oligodendrocytes were generated within three months from monkey embryonic stem cells. The embryonic stem cell-derived oligodendrocytes exhibited *in vitro* myelinogenic potency with rat dorsal root ganglion neurons. Additionally, the transplanted oligodendrocyte progenitor cells differentiated into myelin basic protein-positive mature oligodendrocytes in the mouse corpus callosum. This preparative method was used for human induced pluripotent stem cells, which were also successfully differentiated into oligodendrocyte progenitor cells and mature oligodendrocytes that were capable of myelinating rat dorsal root ganglion neurons. Moreover, it was possible to freeze, thaw, and successfully re-culture the differentiating cells. These results showed that embryonic stem cells and human induced pluripotent stem cells maintained in a dissociated monolayer and feeder-free culture system have the potential to generate oligodendrocyte progenitor cells and mature oligodendrocytes *in vitro* and *in vivo*. This culture method could be applied to prepare large amounts of oligodendrocyte progenitor cells and mature oligodendrocytes in a relatively short amount of time.

## Introduction

Pluripotent stem cells (PSCs), including embryonic stem cells (ESCs) and induced pluripotent stem cells (iPSCs), have the potential to differentiate into several types of functional cells. Therefore, primate PSCs are believed to be extremely versatile cells that could be used for *in vitro* models for drug evaluation and *in vivo* use for regenerative medicine. However, there are still some problems regarding practical use. One of the technical challenges is the complexity of PSC manipulation. PSCs are conventionally disseminated on feeder cells to form an adequate colony. However, it is too complicated to consistently maintain the cells in an undifferentiated state and to differentiate the cells into desired functional cells. To address these problems, previous studies have developed alternative methods, such as feeder-free and dissociated monolayer culture systems [1, 2]. In these reports, the PSCs were maintained in undifferentiated states, expressed many pluripotency markers, and had the differentiation capacity by teratoma formation. However, no study has concluded whether these PSCs had the potential to differentiate into multiple types of functional cells. We previously reported a dissociated monolayer and feeder-free culture system for primate PSCs using cynomolgus monkey ESCs, CMK6_SFF_, and CMK970 [3]. Using this culture system, ESCs were passaged many times and propagated at a high proliferation rate, while retaining the typical ESC properties. Furthermore, these cells were able to differentiate into cortical neurons.

There is great focus on the development of novel therapeutic strategies to treat central nervous system damage with demyelination being one of the biggest challenges [4–7]. The promotion of remyelination is an ideal therapeutic strategy to provide protection from further damage and demyelination [8, 9]. Methods that employ *in vitro* myelinating systems with differentiated human oligodendrocytes could serve as a powerful tool for the development of drugs to promote remyelination.

Recently, several groups have shown that oligodendrocyte progenitor cells (OPCs) and oligodendrocytes could be generated from human iPSCs cultured as a colony and with or without feeder cells [10–14]. However, very little is known about the derivation of OPCs from human iPSCs, which are maintained in dissociated monolayer culture systems. The dissociated monolayer and feeder-free culture system has the advantage of being able to prepare large quantities of OPCs. For the establishment of *in vitro* assay systems, it is essential to be able to store frozen differentiated OPCs, because it takes more than two months to obtain OPCs from undifferentiated cells. The lack of efficient methods to store frozen cells inhibits the clinical application of human iPSCs.

In this report, we developed a differentiation method from monkey ESCs and human iPSCs using the dissociated monolayer and feeder-free culture system to prepare large-scale cryopreserved and functional OPCs for drug discovery and regenerative medicine.

## Materials and methods

### Differentiation from monkey ESCs

#### Preculture (Step 0)

The care and experimental procedures of cynomolgus monkeys were in accordance with a protocol approved by the Animal Care and Use Committee of Shiga University of Medical Science [Permit Number: 2011-10-5H]. Mature cynomolgus monkeys were housed individually in cages that were 500 mm wide by 800 mm deep by 800 mm high. Light cycle was 12 h of artificial light from 8:00 to 20:00. Temperature and humidity in the animal room were maintained at 25±2°C and 50±5%, respectively. Each animal was fed 20 g/kg/day of commercial pellet monkey chow (CMK-1, CLEA, Japan) in the morning, supplemented with 20–50 g of sweet potato and half a banana in the afternoon. Water was supplied ad libitum by an automatic supplier. No monkeys were sacrificed in this study. The animal welfare and steps taken to ameliorate suffering were in accordance with the recommendations of the Weatherall report, “The use of non-human primates in research”. The cynomolgus monkey ESCs, CMK6_SFF_ and CMK970, were established and maintained as previously described [3]. Briefly, these cells were grown in MT-fCFA medium [3]. The cells were dissociated into single cells with 0.005% trypsin/0.002% EDTA (Sigma-Aldrich) and plated on collagen type I-coated dishes (IWAKI) with MT-fCFA medium containing 2.4 μM thiazovivin (Wako) and 4.7 μg/mL human fibronectin (Corning). The cells were further cultured in MT-fCFA medium, which was replaced with fresh medium every day.

#### Induction into Neuroectodermal (NE) cells (Step 1)

The undifferentiated cynomolgus monkey ESCs, CMK6_SFF_ and CMK970, were plated on collagen type I-coated dishes and treated with 10 μM SB431542 (Wako), 0.1 μM LDN193189 (Stemgent), 10 μM Y-27632 (Wako), and 5 μM human fibronectin in MT-CDM medium [3]. The cells were cultured for 3 days and treated in MT-CDM medium for 1 day, followed by 10 μM all-trans retinoic acid (RA; Stemgent) and 200 ng/mL Sonic Hedgehog (SHH; R&D Systems) for 2 days.

#### Differentiation into OPCs (Step 2)

The NE cells from monkey ESCs were detached with TrypLE Select (Thermo Fisher Scientific) and suspended in Dulbecco’s Modified Eagle Medium (DMEM)/F12 (Thermo Fisher Scientific) supplemented with 20 ng/mL FGF2, 200 ng/mL SHH, 20 ng/mL PDGF-AA (R&D Systems), 1 × B-27 Supplement without vitamin A (Thermo Fisher Scientific), 1 × N-2 Supplement (Thermo Fisher Scientific), 100 units/mL penicillin, and 100 μg/mL streptomycin (Sigma-Aldrich), and were then allowed to grow in suspension as spheres for 44 days. The medium was changed twice weekly. The spheres were treated with 1 × Accutase (Thermo Fisher Scientific) for 5 min and centrifuged. The cell pellet was resuspended with the same medium and dissociated into single cells by pipetting. The cells were transferred to plates coated with 0.01% poly-L-ornithine (Sigma-Aldrich) and 10 μg/mL laminin (Sigma-Aldrich) (poly-L-ornithine/laminin) and cultured for 7 days. The medium was changed twice weekly. The cells were detached with Accutase and centrifuged. The cells were suspended in STEM-CELLBANKER (Takara Bio) and cryopreserved in -80°C refrigerator.

#### Differentiation into oligodendrocytes *in vitro* (Step 3)

After 581 and 1006 days (CMK6 _SFF_), or 1183 days (CMK970) of storage, the cryopreserved cells were thawed and plated onto 24-well poly-L-ornithine/laminin-coated culture plates in DMEM/F12 supplemented with 20 ng/mL FGF2, 200 ng/mL SHH, 20 ng/mL PDGF-AA, 1 × B-27 Supplement without vitamin A, 1 × N-2 Supplement, 100 units/mL penicillin, and 100 μg/mL streptomycin at 2.5 × 10^5^ cells per well. After 1 or 2 days, the medium was removed and replaced with DMEM/F12 with 60 ng/mL 3,3′,5-triiodo-L-thyronine (T3; Sigma-Aldrich), 100 μM dibutyryl cyclic-AMP sodium salt (dbcAMP; Sigma-Aldrich), 200 ng/mL SHH, 100 ng/mL Noggin (R&D Systems), 100 ng/mL insulin-like growth factor-I (IGF-1; R&D Systems), 10 ng/mL neurotrophin-3 (NT-3; R&D Systems), 1 × B-27 Supplement without vitamin A, 1 × N-2 Supplement, 100 units/mL penicillin, and 100 μg/mL streptomycin. Half of the media were changed twice weekly.

### Differentiation from human iPSCs

#### Preculture (Step 0)

The human iPSCs, F4V3-A3 (A3) from human dermal fibroblasts (Cell Applications) and #1–04 from human peripheral blood mononuclear cell (Cellular Technology Limited), were maintained the same as the monkey ESCs described in Step 0.

#### Induction into NE cells (Step 1)

The undifferentiated human iPSCs (A3 and #1–04) were plated on collagen type I-coated dishes with MT-fCFA medium containing 2.4 μM thiazovivin and 4.7 μg/mL human fibronectin. The cells were treated with 10 μM SB431542 and 0.3 μM LDN193189 in MT-CDM medium the next day. After 3 days, the media were changed and the cells were cultured for another 3 days. The cells were incubated in MT-CDM medium for 1 day, followed by MT-CDM with 10 μM RA and 200 ng/mL SHH for 2 days.

#### Differentiation into OPCs (Step 2)

The cells were treated similar to the monkey cells, but grown in suspension as spheres for 67 days. The dissociated cells were cultured for 8 days.

#### Differentiation into oligodendrocytes *in vitro* (Step 3)

The procedure was the same as that for the monkey cells. The cryopreserved cells that were stored for 30 and 90 days (A3), or 15 days (#1–04) were used.

### Immunocytochemistry

Cells were fixed with 4% paraformaldehyde for 10 min and rinsed three times with phosphate-buffered saline (PBS). The cells were permeabilized with 0.1% Triton X-100 for 15 min and blocked with PBS containing 1% normal donkey serum, 0.1% Triton X-100 and 1% bovine serum albumin (BSA) for 1 hour. Primary antibodies were incubated overnight at 4°C. To label O4 and PDGFRα, the cells were incubated with primary antibody and 1% normal donkey serum for 20 min before fixation. Appropriate Alexa Fluor-conjugated secondary antibodies (1:500; Thermo Fisher Scientific) were incubated for 1 hour. The nuclei were counterstained with 20 μM Hoechst 33342 (Dojindo) for 7 min, and the cells were rinsed three times with PBS. The primary antibodies were as follows: rabbit monoclonal anti-Nanog (1:800; Cell Signaling Technology, 4903), mouse monoclonal anti-SSEA-4 (1:100; Stemgent, 09–0006), goat polyclonal anti-Oct4 (1:100; Santa Cruz, sc-8628), mouse monoclonal anti-Sox2 (1:100; R&D Systems, MAB2018), goat polyclonal anti-Sox1 (1:100; R&D Systems, AF3369), mouse monoclonal anti-Pax6 (1:200; BD Biosciences, 561462), rabbit polyclonal anti-Olig2 (1:100; EMD Millipore, AB9610), mouse monoclonal anti-Nkx2.2 (1:50; DSHB, 74.5A5), mouse monoclonal anti-PDGFRα (1:50; BD Biosciences, 556001), mouse monoclonal anti-A2B5 (1:100; Sigma-Aldrich, A8229), mouse monoclonal anti-O4 (1:50; R&D Systems, MAB1326), rat monoclonal anti-MBP (1:50; EMD Millipore, MAB386), rabbit polyclonal anti-GFAP (1:10000; EMD Millipore, AB5804), mouse monoclonal anti-S100β (1:200; Abcam, ab66028), mouse monoclonal anti-HB9 (1:50, DSHB, 81.5C10), and rabbit monoclonal anti-β-III tubulin (1:1000, Covance, MRB-435P) antibody. The secondary antibodies were as follows: Alexa Fluor 488 or 555 donkey anti-rabbit IgG (H+L), Alexa Fluor 488 donkey anti-mouse IgG (H+L), Alexa Fluor 555 donkey anti-goat IgG (H+L), Alexa Fluor 555 goat anti-rat IgG (H+L), and Alexa Fluor 488 goat anti-mouse IgM heavy chain. The fluorescence images were captured with a fluorescence microscopy system (DeltaVision Elite Imaging System; GE Healthcare). Quantitative evaluation of O4- and/or MBP-positive cells was performed using an IN Cell Analyzer 2200 microscope and IN Cell Developer Toolbox software (GE Healthcare). Data are normalized by nuclei number.

### Surgical procedures for CMK6_SFF_ cell transplantation

The care and experimental procedures of all animals were in accordance with a protocol approved by the Institutional Animal Care and Use Committee of the Asahikawa Medical University [Permit Number: 14082, 15016 and 16012]. After 499 days, cryopreserved OPCs differentiated from CMK6_SFF_ were thawed and allowed to recover for 1 week prior to surgery by plating on poly-L-ornithine/laminin-coated culture plates in DMEM/F12 supplemented with 20 ng/mL FGF2, 200 ng/mL SHH, 20 ng/mL PDGF-AA, 1 × B-27 Supplement without vitamin A, 1 × N-2 Supplement, 100 units/mL penicillin, and 100 μg/mL streptomycin. Pups obtained from C57BL6/J mice (Sankyo Labo Service) were anesthetized by hypothermia, and 1 × 10^5^ cells in 1 μL were injected in the cerebral cortex (Cx) at a depth of 1–1.2 mm into the corpus callosum (CC) of postnatal day 2–3 pups [13]. Cells were injected though a Hamilton syringe. Totally 9 mice were used. We checked the number of animals and monitored their size, body weight and behavior including eating and drinking every day. In case the animals showed either severe motor dysfunction such as paralysis or abnormal behavior, we had a protocol for humane experimental endpoints for animals. However, we did not have any animals who became severely ill or died. During the experiments, the animals looked healthy/normal and they did not show any motor dysfunction or abnormal behavior. There were no animals who became ill after transplantation. Although we actually did not need to perform the early euthanasia, we had a protocol in place for it. When the animals cannot recover well or get more severe illness/moribund by misfortune, we would decide to use overdose of euthanasia (mixture of medetomidine/butorphanol/midazolam).

### Immunohistochemistry

Animals were sacrificed with a lethal dose of anesthetic (mixture of medetomidine/butorphanol/midazolam) at 4 weeks and the Cx was frozen using the snap-freezing method with dry ice. All efforts were also made to minimize suffering. Fresh-frozen coronal sections from the mouse brain (14 μm) were cut on a cryostat and stored at −30°C until further use. Transplanted CMK6_SFF_ was identified with mouse monoclonal anti-human nuclei (HuN) antibody (1:250; Takara Bio, STEM101). The sections were also stained with mouse monoclonal anti-MBP (1:5000; EMD Millipore, SMI-94) or human-specific mouse monoclonal anti-GFAP (1:200, Takara Bio, STEM123) antibody. Immunostaining was performed following a standard fluorescein protocol [15]. Briefly, the sections were blocked with 2% normal goat serum, 5% BSA, and 0.2% Triton X-100, and then incubated with primary antibodies at 4°C overnight. Alexa Fluor 488 or 568 goat anti-mouse IgG (H+L) (1:1000) were used to visualize the primary antibodies. The sections were analyzed with a confocal laser microscope (FV-1000D, Olympus) and software (Fluoview, Olympus). Each group included tissue sections from three to four animals.

### Co-culture of PSC-derived OPCs with rat primary Dorsal Root Ganglion (DRG) neurons *in vitro*

The care and experimental procedures of all animals were in accordance with a protocol approved by the Japanese National Research Institute for Child Health and Development Animal Care Committee [Permit Number: 2002–005, 2002–006 and 2005–005]. Pregnant rats were sacrificed with overdose of sodium pentobarbital (100 mg/kg body weight). DRG neurons were isolated from E15 SD rat spinal cord regions as previously described [16] and then dissociated and plated onto collagen type I-coated coverslips (IWAKI). Non-neuronal cells were eliminated by cycling them three times with medium containing 5-fluorodeoxyuridine and uridine. Myelinating co-cultures were established by seeding approximately 2 × 10^5^ differentiated monkey ESCs or human iPSCs onto purified DRG neurons as previously described [17]. The cryopreserved monkey ESCs or human iPSCs-derived OPCs that were stored for 131 or 60 days were used. Co-cultures were maintained and medium was replaced every 3 days.

Co-cultures were fixed first with 4% paraformaldehyde and then with 100% cold methanol [18]. The fixed cells were permeabilized with PBS containing 0.1% Tween-20 and blocked using the Blocking One kit (Nacalai Tesque). The cells were incubated first with primary antibodies and then with appropriate Alexa Fluor-conjugated secondary antibodies. The coverslips or dishes were mounted with Vectashield reagent containing 4′,6-diamidino-2-phenylindole (DAPI; Vector Laboratories, Burlingame, CA). Primary antibodies were as follows: mouse monoclonal anti-MBP (1:100; BioLegend, SMI-94R) and rabbit polyclonal anti-neurofilament 200 (1:100; Sigma-Aldrich, N4142) antibody. The secondary antibodies were as follows: Alexa Fluor 488 goat anti-rabbit IgG (H+L), and Alexa Fluor 594 donkey anti-mouse IgG (H+L). The fluorescence images were captured with a fluorescence microscopy system (DMI4000B; Leica, DeltaVision Elite Imaging System; GE Healthcare) and analyzed with AF6000 software (Leica).

### Western blotting analysis

Cells were lysed in lysis buffer (50 mM HEPES-NaOH, pH 7.5, 20 mM MgCl_2_, 150 mM NaCl, 1 mM dithiothreitol, 1 mM phenylmethanesulfonyl fluoride, 1 mg/ml leupeptin, 1 mM EDTA, 1 mM Na_3_VO_4_, and 10 mM NaF) containing detergents (0.5% NP-40, 1% CHAPS, and 0.1% SDS). These detergents are important for myelin protein isolation [18, 19]. Unless otherwise indicated, all steps were performed at 4°C. Equal amounts of protein (20 μg total protein) in centrifuged cell supernatants were heat-denatured and prepared for immunoblotting using the TransBlot TurboTransfer System (Bio-Rad). The transferred membranes were blocked with a Blocking One kit and incubated with mouse monoclonal anti-MBP (BioLegend, SMI-94R) and mouse monoclonal anti-CNPase (Sigma-Aldrich, C5922) antibodies, followed by a peroxidase-conjugated secondary antibody. The bound antibodies were detected using a Chemiluminescence ImmunoStar Zeta kit (Wako).

### Gene expression analysis

The cryopreserved human iPSCs-derived OPCs that were stored for 90 days were used. RNA was isolated using the RNeasy Micro Kit (Qiagen), and cDNA was synthesized using the High-Capacity cDNA Reverse Transcription Kit (Thermo Fisher Scientific) according to the manufacturer’s protocols. Subsequently, qPCR was performed using the TaqMan Universal Master Mix II with UNG and TaqMan Gene Expression Assays (Thermo Fisher Scientific). The qPCR reaction was performed on the QuantiStudio 7 Flex Real-Time PCR System (Thermo Fisher Scientific) according to the manufacturer’s protocol. TaqMan Gene Expression Assays were as follows: MBP (*MBP*, Hs00921945_m1), PLP1 (*PLP1*, Hs00166914_m1), CLDN11 (*CLDN11*, Hs00194440_m1), and 18S (*18S*, Hs99999901_s1). Gene expression values were adjusted for 18S expression values.

### Flow cytometry

The cells that were harvested using Accutase were suspended in PBS containing 1% fetal bovine serum, 0.1% NaN_3,_ and PE-conjugated mouse IgG2a, κ Isotype control (BD Biosciences, 555574) or mouse monoclonal anti-PDGFRα antibody (BD Biosciences, 556002). The cells were put on ice for 30 min and were washed. Flow cytometry were performed on a BD LSRFortessa cell analyzer (BD Biosciences) and the data were analyzed FlowJo software (FlowJo).

## Results

### OPCs and oligodendrocytes are successfully differentiated from monkey ESCs maintained in the dissociated monolayer and feeder-free culture system

**Fig 1:**
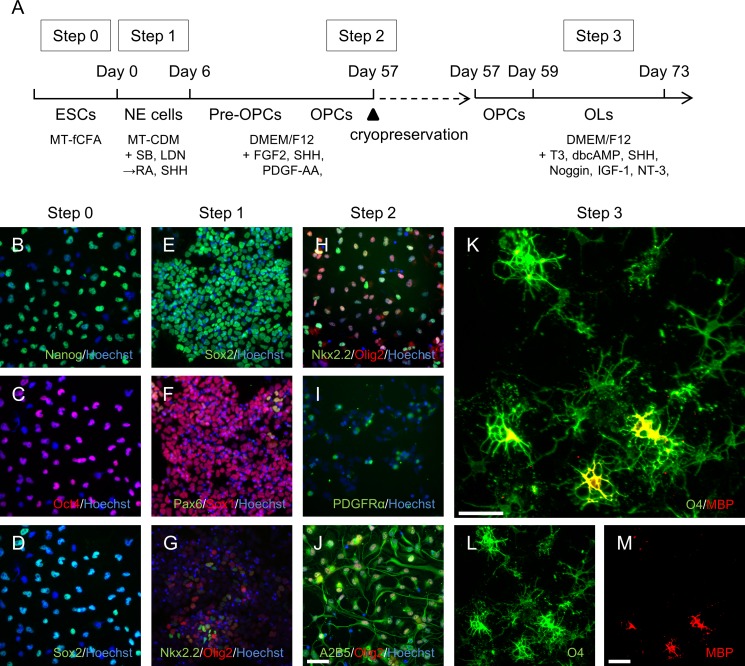
OPCs and oligodendrocytes derived from the monkey ESCs CMK6_SFF._ (A) Protocol for differentiation into OPCs and oligodendrocytes. (B–D) Immunostained CMK6_SFF_ before differentiation. Cultured cells in Step 0 immunostained with Nanog (green) and Hoechst (blue, B). Cells stained with Oct4 (red) and Hoechst (blue, C). Sample from panel C also stained with Sox2 (green, D). (E–G) Immunostained CMK6_SFF_ from Step 1. Cells from day 6 immunostained with Sox2 (green) and Hoechst (blue, E). Cells stained with Pax6 (green), Sox1 (red), and Hoechst (blue, F), as well as Nkx2.2 (green), Olig2 (red), and Hoechst (blue, G). (H–J) Immunostained CMK6_SFF_ from Step 2. Cells were cryopreserved for 581 days and the cells from day 59 immunostained with Nkx2,2 (green), Olig2 (red), and Hoechst (blue, H). Cells stained with PDGFRα (green) and Hoechst (blue, I), as well as with A2B5 (green), Olig2 (red), and Hoechst (blue, J). (K–M) Immunostained CMK6_SFF_ from Step 3. Cells from day 73 immunostained with O4 (green) and MBP (red). Scale bars = 50 μm.

Because there is no established protocol for generating oligodendrocytes from monkey ESCs, we modified several published protocols on human PSCs [10, 12] and mouse epiblast stem cells [20]. The monkey ESCs, CMK6_SFF_, were maintained in the proprietary dissociated monolayer and feeder-free culture system. To create a broadly applicable protocol for oligodendrocyte differentiation, we divided the method into three steps to optimize the respective protocols and then combined them at the end (Fig 1A).

For the first step, the undifferentiated CMK6_SFF_ was directed toward NE cells with SB431542, an inhibitor of activin-nodal signaling, and LDN193189, an inhibitor of BMP signaling, for 3 days. After culturing for 1 day in medium without supplements, the medium was replaced with medium containing RA to help determine positions along the embryonic anterior/posterior axis and SHH, which served as a ventralizing factor, because OPCs arise as NE cells in the ventral ventricular zone [21]. During this time, expression of pluripotency markers Nanog (Fig 1B) and Oct4 (Fig 1C) was completely and immediately diminished, while expression of the pluripotency and neuroectoderm progenitor marker Sox2 remained (Fig 1D and 1E). The NE marker Sox1 was expressed and Pax6 also emerged (Fig 1F). The oligodendroglial lineage markers Nkx2.2 and Olig2 were also expressed (Fig 1G).

For the second step, the NE cells were differentiated into OPCs in the presence of FGF2 and PDGF-AA, which induce proliferation of OPCs [22, 23]. The cells had to first form sphere clusters for 44 days to differentiate into OPCs over a short period of time. After dissociation of the spheres into single cells and culturing them for 7 days, the proportion of late pre-OPC marker Nkx2.2-positive cells increased, resulting in augmentation of Olig2-Nkx2.2 double-positive gliogenic cells (Fig 1H). These cells also expressed the OPC marker PDGFRα and the glial progenitor marker A2B5 (Fig 1I and 1J).

For the third step, OPC proliferation factors were removed from the culture medium, and the cells were treated with T3 to induce oligodendrocyte differentiation. At 14 days post-differentiation, the cells expressed the oligodendrocyte cell-surface marker O4 and multiple processes appeared. Moreover, some of these cells also started to express the mature oligodendrocyte marker MBP. The results showed that oligodendrocytes were successfully differentiated from undifferentiated CMK6_SFF_. We also confirmed that this protocol was applicable with the additional monkey ESCs, CMK970 (S1 Fig).

We furthermore addressed the possibility of cryopreservation to prepare the same quality of oligodendrocytes during any time of need. At the end of Step 2, OPCs were frozen using the STEM-CELLBANKER, and cryopreserved OPCs were thawed and successfully differentiated into oligodendrocytes using a similar process for 14 days (Fig 1K–1M). Results showed that the quality was the same as for cells without cryopreservation. Therefore, it was concluded that this differentiation method was relatively stable, and the cryopreserved OPCs were used for further experiments.

### CMK6_SFF_-derived OPCs differentiate into MBP-expressing oligodendrocytes in mouse brain

**Fig 2:**
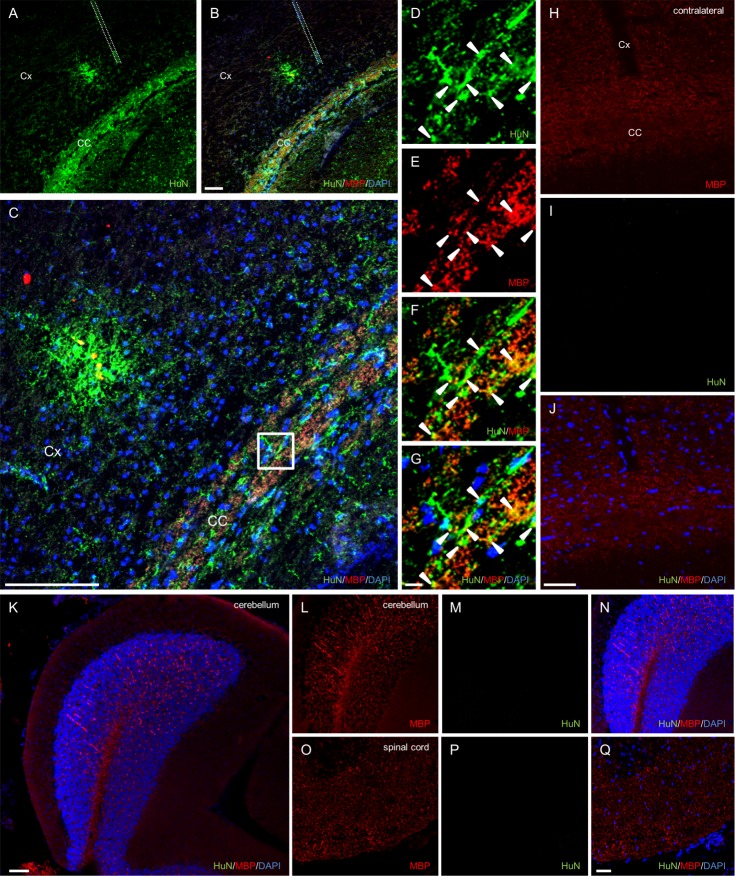
Transplanted CMK6_SFF_-derived OPCs differentiate into oligodendrocytes *in vivo*. Distribution of CMK6_SFF_-derived cells transplanted into the corpus callosum (CC) of the cerebral cortex (Cx) in neonatal mice. (A) Sections stained with HuN (green) in the CC and Cx (B). The same section immunostained with MBP (red) and DAPI (blue). (C) Enlarged image of B. The dotted lines show needle insertion locations. Scale bars = 50 μm. (D-G) High-magnification images with boxed region in C. Arrowheads show HuN-positive cells co-stained with MBP. Scale bars = 10 μm. (H-J) Immunostained contralateral cerebral cortex with MBP (red), HuN (green) and DAPI (blue). (K) Immunostained cerebellum sections. Enlarged images of MBP (L), HuN (M), and overlay (N). Spinal cord section (O–Q). Scale bars = 50 μm.

To assess whether CMK6_SFF_-derived OPCs were functionally myelinogenic *in vivo*, we injected CMK6_SFF_-derived OPCs into the CC of the Cx in mouse neonates. The animals were sacrificed at 4 weeks after injection. Immunohistochemistry revealed HuN-positive cells mainly in the CC and partly in the Cx of recipient animals (Fig 2A–2G). In the CC, many HuN-positive cells also expressed MBP, suggesting that the implanted cells differentiated into mature oligodendrocytes. Conversely, HuN-positive cells were not observed in the contralateral cerebral cortex (Fig 2H–2J), cerebellum (Fig 2K–2N) or spinal cord (Fig 2O–2Q), although MBP was identified. These results suggested that CMK6_SFF_-derived OPCs are capable of mature oligodendrocyte differentiation *in vivo*.

### OPCs derived from monkey ESCs exhibit myelinogenic potential *in vitro*

**Fig 3:**
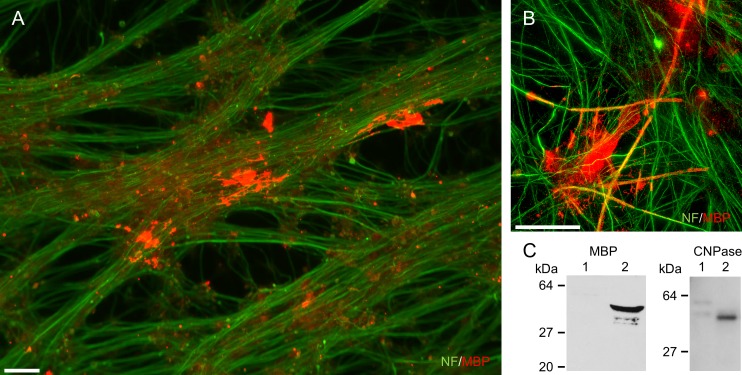
CMK6_SFF_-derived OPCs exhibit myelinogenic potency *in vitro*. OPCs from monkey ESCs co-cultured with rat primary DRG neurons for 45 days. (A, B) Neurons and differentiated oligodendrocytes immunostained with NF (green) and MBP (red), respectively. Scale bars = 30 μm. (C) Western blotting analysis of MBP protein and CNPase protein in rat primary DRG neurons without (lane 1) or co-cultured with OPCs (lane 2).

The processes of DRG neurons can be myelinated by oligodendrocytes *in vitro* [17]. In the current study, to evaluate myelinogenic function *in vitro*, CMK6_SFF_-derived OPCs were cultured with rat primary DRG neurons. The differentiated oligodendrocytes were attached to axons and co-stained with several DRG neurons after 45 days (Fig 3A and 3B). The oligodendrocyte markers MBP protein and CNPase protein were detected in the co-culture (Fig 3C). These results suggested that monkey ESC-derived oligodendrocytes exhibit myelinogenic function *in vitro*.

### Functional OPCs and oligodendrocytes are successfully induced from human iPSCs maintained in the dissociated monolayer and feeder-free culture system

**Fig 4:**
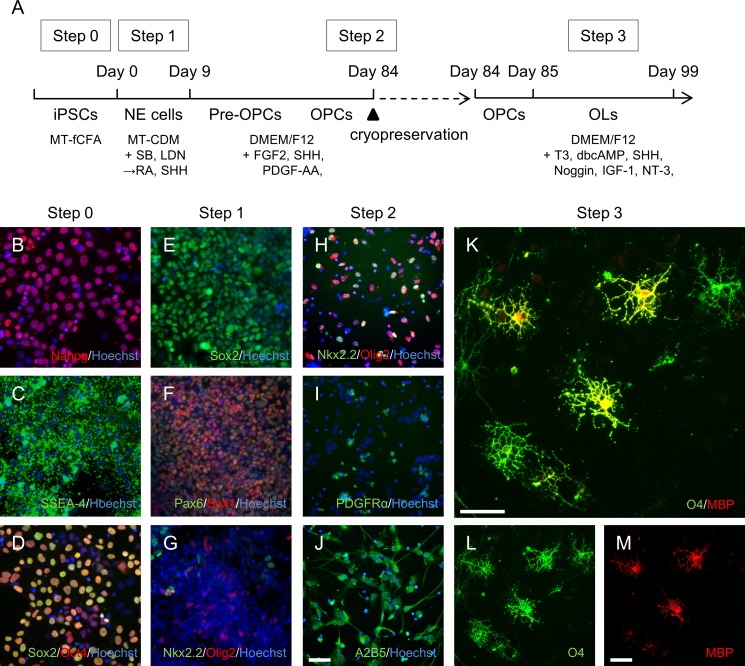
OPCs and oligodendrocytes derived from the human iPSCs A3. (A) Protocol for differentiation into OPCs and oligodendrocytes. (B–D) Immunostained A3 prior to differentiation. Cultured cells from Step 0 immunostained with Nanog (red) and Hoechst (blue, B), as well as with SSEA-4 (green) and Hoechst (blue, C). Cells stained with Sox2 (green), Oct4 (red), and Hoechst (blue, D). (E–G) Immunostained A3 from Step 1. Cells from day 9 immunostained with Sox2 (green) and Hoechst (blue, E). Cells stained with Pax6 (green), Sox1 (red), and Hoechst (blue, F), as well as with Nkx2.2 (green), Olig2 (red), and Hoechst (blue, G). (H–J) Immunostained A3 from Step 2. Cells were cryopreserved for 90 days and the cells from day 85 immunostained with Nkx2,2 (green), Olig2 (red), and Hoechst (blue, H). Cells stained with PDGFRα (green) and Hoechst (blue, I), as well as with A2B5 (green), and Hoechst (blue, J). (K–M) Immunostained A3 from Step 3. Cells from day 99 immunostained with O4 (green) and MBP (red). Scale bars = 50 μm.

**Fig 5:**
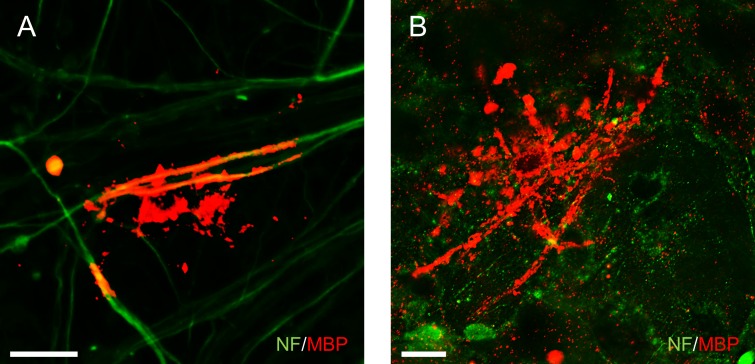
OPCs from A3 exhibit myelinogenic potency *in vitro*. OPCs from human iPSCs were co-cultured with rat primary DRG neurons for 45 days (A) and 70 days (B). Neurons and differentiated oligodendrocytes immunostained with NF (green) and MBP (red), respectively. Scale bars = 20 μm.

We applied our dissociated monolayer and feeder-free culture system to existing human iPSCs and postulated that the new human iPSCs, A3, exhibit features similar to monkey ESCs. A3 can be passaged many times by single-cell dissociation using traditional trypsin treatments; the cells can be propagated as a monolayer without feeder cells with a high proliferation rate. Therefore, we applied the oligodendrocyte differentiation method to the A3. In this protocol (Fig 4A), Nanog, SSEA-4, Sox2, and Oct4-positive undifferentiated iPS cells were directed toward the Sox2, Pax6, and Sox1-positive NE cells, which also expressed Olig2 or Nkx2.2 markers at Step 1 (Fig 4B–4G). At the end of the second step, Olig2-Nkx2.2 double-positive, as well as PDGFRα- and A2B5-positive cells, were cryopreserved (Fig 4H–4J). The OPC surface marker PDGFRα was also determined by flow cytometry (S2 Fig). For oligodendrocyte generation, thawed cells were cultured with oligodendrocyte maturation factors. Consequently, O4- and MBP-positive oligodendrocytes appeared within 99 days after initiation of differentiation (Fig 4K–4M). Expression of the oligodendrocyte-related genes MPB, PLP1 and CLDN11 was upregulated over time (S3 Fig). The other human iPSCs, #1–04, also differentiated into OPC and O4- and/or MBP-positive oligodendrocytes using the same protocol as for the A3 cells (S4 Fig). To determine the myelinogenic potency of human iPSC-derived oligodendrocytes, A3-derived OPCs were co-cultured with rat DRG neurons. The oligodendrocytes attached to neural processes from several different neurons at 45 days and formed MBP-positive structures by 70 days (Fig 5A and 5B). These results suggested that human iPSC-derived oligodendrocytes exhibit myelinogenic function. Thus, our protocol to generate OPCs and oligodendrocytes could be broadly applied, and the dissociated monolayer and feeder-free culture system provides a reliable platform for maintaining primate PSCs.

## Discussion

Several groups have reported that OPCs and oligodendrocytes could be generated from human iPSCs, which are cultured as a colony with or without feeder cells [10–14]. It is difficult to obtain large amounts of OPCs, because most culture systems have problems with quality control and colony growth. We previously established the dissociated monolayer and feeder-free culture system for primate PSCs and showed that this system was useful for inducing differentiation of functional neurons, indicating that our dissociated monolayer and feeder-free culture system could consistently produce large amounts of functional cells [3]. In the present study, we generated functional OPCs and oligodendrocytes from two monkey ESCs, CMK6_SFF_ and CMK970, and from two human iPSCs, A3 and #1–04. Our results suggested that the dissociated monolayer and feeder-free culture system could potentially provide large-scale functional OPCs. Because the mouse epiblast stem cells phenotype is known to be similar to human ESCs [24, 25], we modified the method to generate oligodendrocytes [20]. The protocol was based on embryonic development, so astrocytes and motor neurons that originated from the fetal motor neuron progenitor (pMN) domain were also detected during Step 3 (S5 Fig). These results indicated that our culture system provided a platform for maintaining primate PSCs, as well as differentiation into various types of cells with characteristic features of differentiated cells.

For the establishment of *in vitro* assay systems, it will be essential to store differentiated OPCs in frozen conditions, because it takes >2 months to obtain OPCs from undifferentiated cells. The lack of frozen storage methods inhibits the clinical application of human iPSCs. We cryopreserved OPCs at the end of Step 2, and oligodendrocytes were generated within 14 days from thawed OPCs. The monkey ESCs and human iPSCs-derived cryopreserved OPCs showed the unchanged oligodendrocyte differentiating potency regardless of the period of cryopreservation (S6 Fig). The cryopreserved OPCs were used as a cell source for transplantation and the *in vitro* myelination assay, and the myelinating function of these OPCs was confirmed in this study. Our future goal is to develop therapeutic drugs and regenerative medicines to intervene with demyelinating conditions in the central nervous system. Cryopreserved OPCs could serve as sources for drug screening systems and cell therapy in myelin-related disorders.

The myelinogenic potency of human iPSC-derived OPCs *in vivo* has been previously shown in myelin-deficient and immunodeficient shiverer/rag2 mice [10, 13, 14]. In the present study, monkey ESC-derived OPCs were engrafted into wildtype newborn mice. Results showed differentiation into MBP-positive oligodendrocytes and GFAP-positive astrocytes *in vivo* (S7 Fig), similar to results from *in vitro* differentiation. Because the transplanted OPCs were functionally myelinogenic, these results suggest that this transplantation method could be useful for evaluating the myelinating capacity of differentiated cells.

Although the myelinogenic potency of human iPSC-derived OPCs has been previously demonstrated with co-cultures of human iPSC-derived neurons and human fetal cortical neurons, unambiguous mature myelin formation was not observed [10, 11]. In the present study, OPCs were co-cultured with rat primary DRG neurons, and myelin-like structures were subsequently observed. Rat primary DRG neurons were ensheathed by human fetal OPCs, which form contactin-associated protein and sodium channel protein clustering on axons [26], suggesting that rat DRG neurons could be useful for examining myelinogenic potency *in vitro*. Although further investigations, such as observation by electron microscopy and confirmation of nodes of Ranvier, are required, these results help to establish *in vitro* myelinating models for evaluating the remyelinating capacity of cells.

To evaluate drug efficacy in humans at pre-clinical stages, many groups have attempted to establish human and non-human primate models that reflect pathological conditions [27–30]. The present study is the first to generate OPCs and oligodendrocytes from monkey PSCs. Monkey ESC-derived OPCs pose some advantages: they exhibit similar characteristics to human OPCs and grow much more rapidly than human OPCs. Therefore, monkey ESC-derived OPCs would be suitable cells for analyzing pathology and molecular mechanisms of disease. Furthermore, a human and monkey neuron-oligodendrocyte co-culture system helps us to evaluate efficacy and safety for drugs as well as for donor cells as a regenerative medicine.

## Conclusions

We previously showed differentiation of monkey ESCs, which were maintained in our dissociated monolayer and feeder-free culture system, into cortical neurons. Results from the present study showed the ability of monkey ESCs to differentiate into functional oligodendrocytes. We also generated oligodendrocytes from new human iPSCs, which were maintained in the culture system. These results show that our culture system provides a platform for maintaining primate PSCs, which have the potential to differentiate into various types of cells with characteristic features.

Furthermore, cryopreserved OPCs from monkey ESCs and human iPSCs give us off the shelf cell sources for drug discovery and regenerative medicine. Taken together, OPCs from PSCs maintained in the dissociated monolayer and feeder-free culture system could provide a better understanding about myelin-related disorders and clues for novel treatment strategies.

## Supporting information

S1 Fig Oligodendrocytes derived from the monkey ESCs CMK970. Differentiated CMK970 stained with O4 (green) and MBP (red). Scale bars = 50 μm.
(TIF)

S2 Fig PDGFRα-positive cells from the human iPSCs A3. Flow cytometry analysis of PDGFRα was performed with isotype control antibody (red) or anti-PDGFRα antibody (blue).
(TIF)

S3 Fig Gene expression analysis of oligodendrocyte-related genes. Gene expression analysis was performed with A3-derived OPCs and oligodendrocytes in Step 3. Days 0 and 14 represent days 85 and 99 in Fig 4A, respectively. N.D., not detected.
(TIF)

S4 Fig Oligodendrocytes derived from the human iPSCs #1–04. Differentiated #1–04 stained with O4 (green) and MBP (red). Scale bars = 50 μm.
(TIF)

S5 Fig Astrocytes and motor neurons derived from CMK6_SFF_ *in vitro*. (A–C) CMK6_SFF_-derived cells stained with astrocyte marker S100β (green), GFAP (red), and Hoechst (blue). (D–F) Cells stained with motor neuron marker HB9 (green), β-III tubulin (red), and Hoechst (blue). Scale bars = 50 μm.
(TIF)

S6 Fig Effect of cryopreserved length on oligodendrocyte differentiation potency. (A) CMK6_SFF_-derived OPCs cryopreserved for both 581 days and 1006 days were differentiated into oligodendrocytes. (B) A3-derived OPCs cryopreserved for both 30 days and 90 days were differentiated into oligodendrocytes. O4- and MBP-positive cells (O4+, MBP+) were counted and normalized by nuclei number.
(TIF)

S7 Fig CMK6_SFF_-derived cells differentiate into astrocytes *in vivo*. Distribution of CMK6_SFF_-derived cells transplanted into the corpus callosum of the cerebral cortex in neonatal mice. Sections stained with GFAP (green), MBP (red), and DAPI (blue). Scale bars = 50 μm.
(TIF)
