# Supplementary figures and images for: Differentiation of oligodendrocyte progenitor cells from dissociated monolayer and feeder-free cultured pluripotent stem cells
Source: PLoS One. 2017 Feb 13;12(2):e0171947. doi: 10.1371/journal.pone.0171947 (PMC5305255; doi:10.1371/journal.pone.0171947)

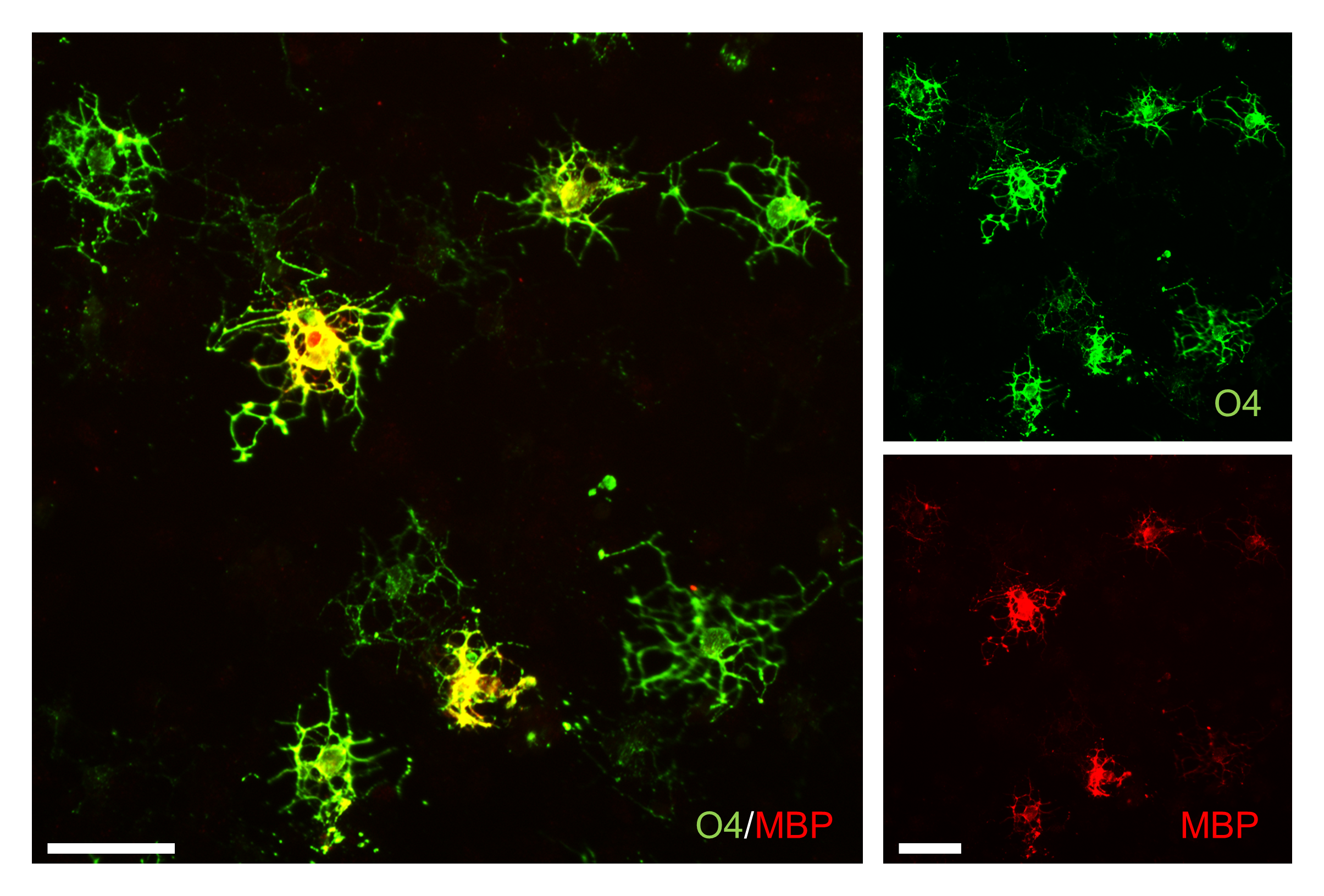

Supplement: S1 Fig — Differentiated CMK970 stained with O4 (green) and MBP (red). Scale bars = 50 μm. (TIF) [file pone.0171947.s001.tif]

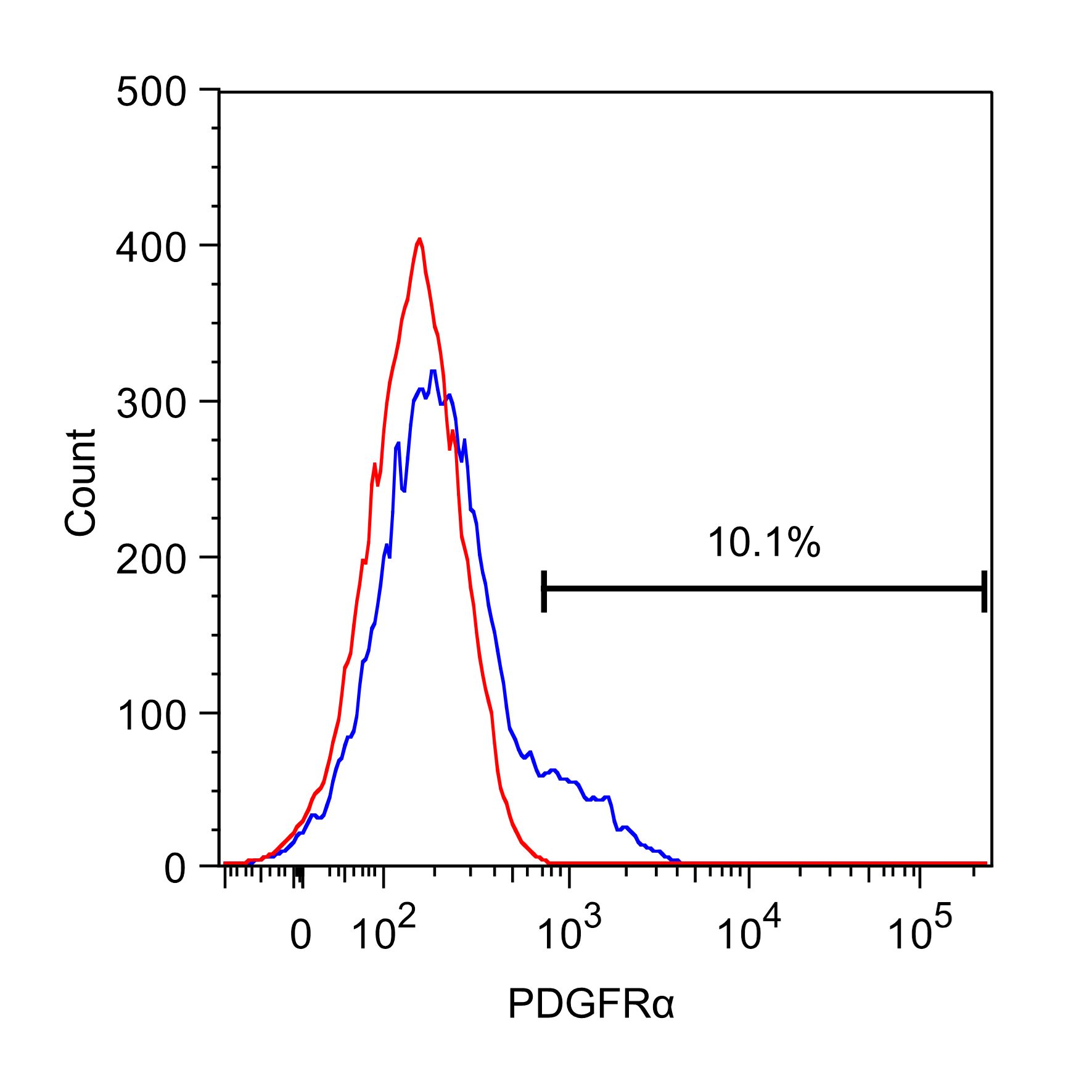

Supplement: S2 Fig — Flow cytometry analysis of PDGFRα was performed with isotype control antibody (red) or anti-PDGFRα antibody (blue). (TIF) [file pone.0171947.s002.tif]

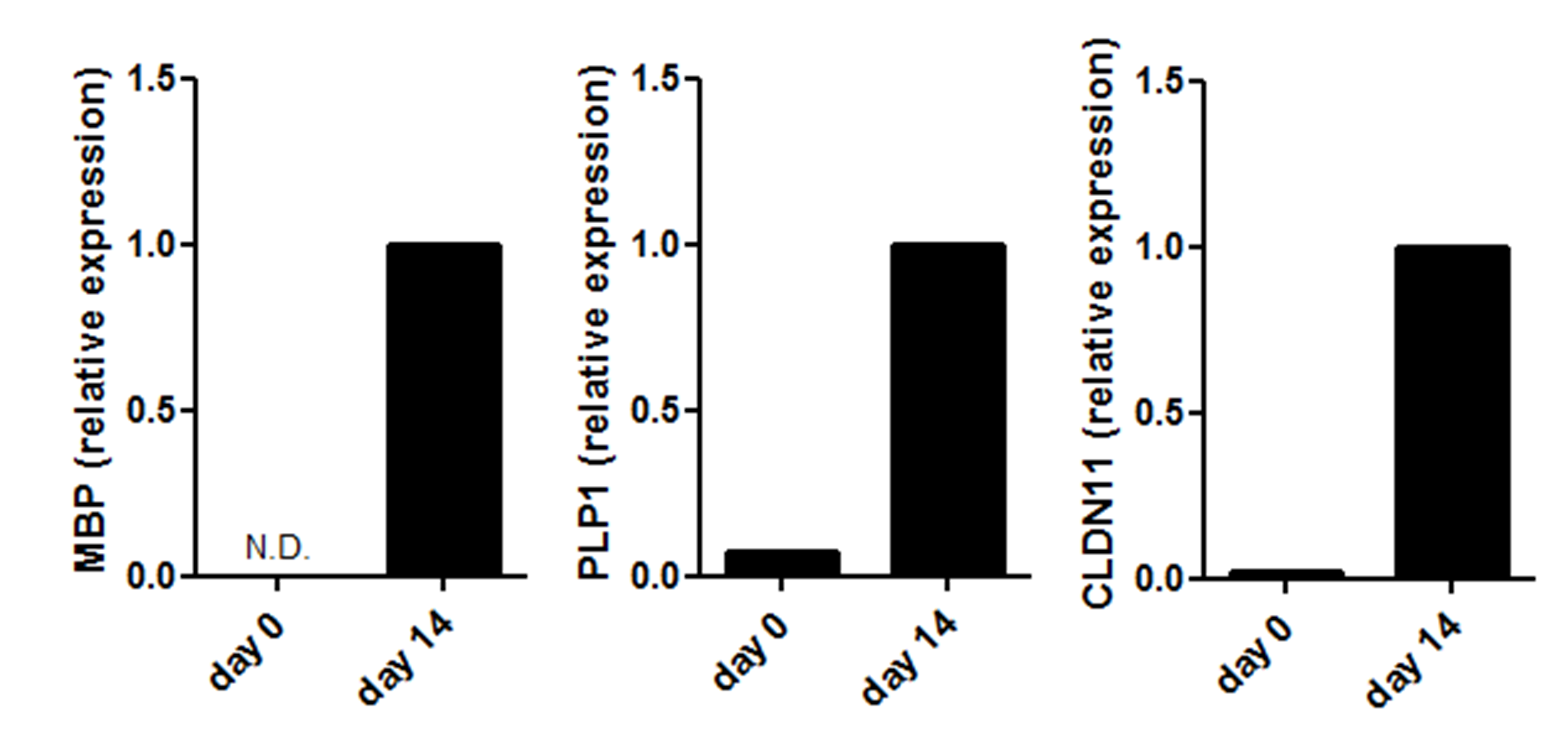

Supplement: S3 Fig — Gene expression analysis was performed with A3-derived OPCs and oligodendrocytes in Step 3. Days 0 and 14 represent days 85 and 99 in Fig 4A, respectively. N.D., not detected. (TIF) [file pone.0171947.s003.tif]

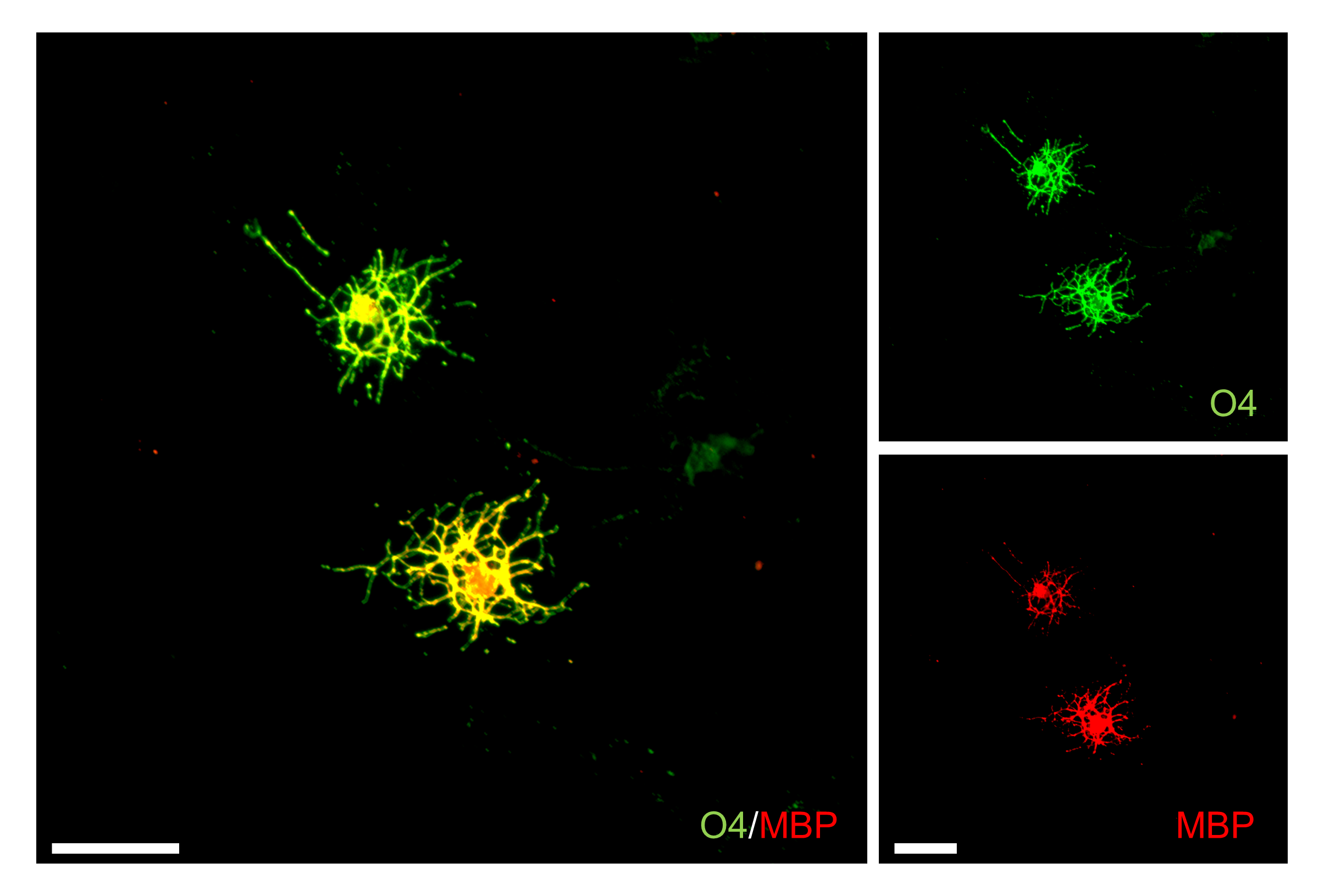

Supplement: S4 Fig — Differentiated #1–04 stained with O4 (green) and MBP (red). Scale bars = 50 μm. (TIF) [file pone.0171947.s004.tif]

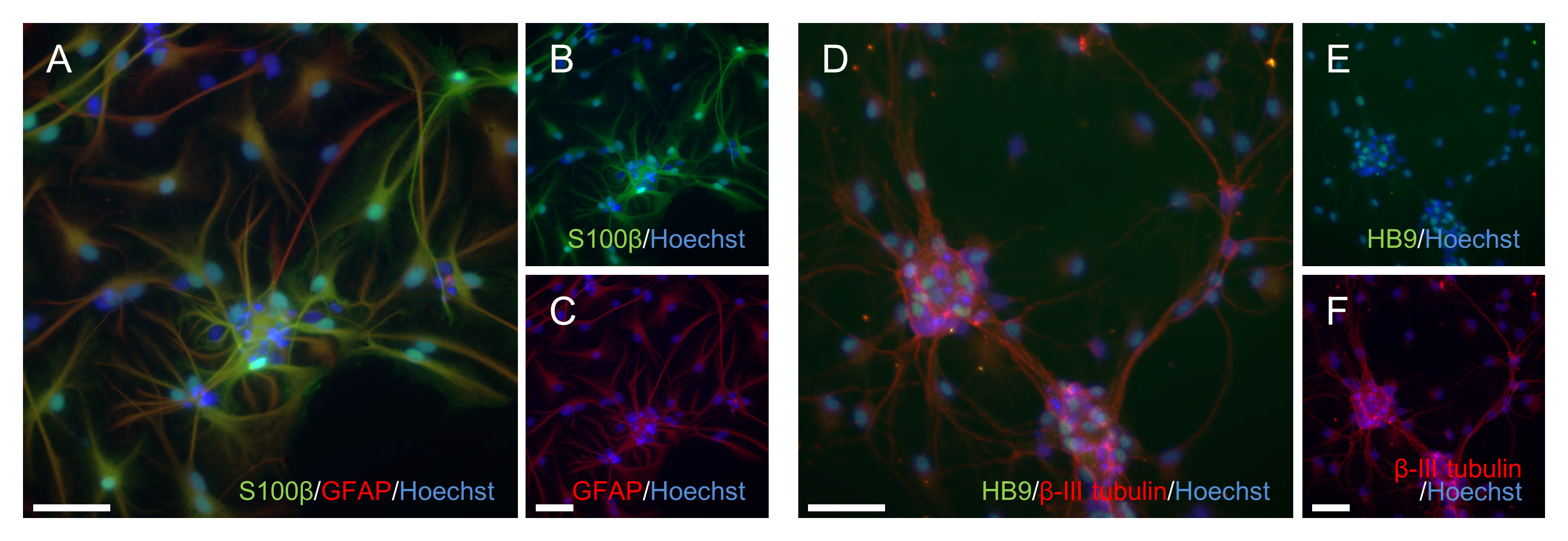

Supplement: S5 Fig — (A–C) CMK6SFF-derived cells stained with astrocyte marker S100β (green), GFAP (red), and Hoechst (blue). (D–F) Cells stained with motor neuron marker HB9 (green), β-III tubulin (red), and Hoechst (blue). Scale bars = 50 μm. (TIF) [file pone.0171947.s005.tif]

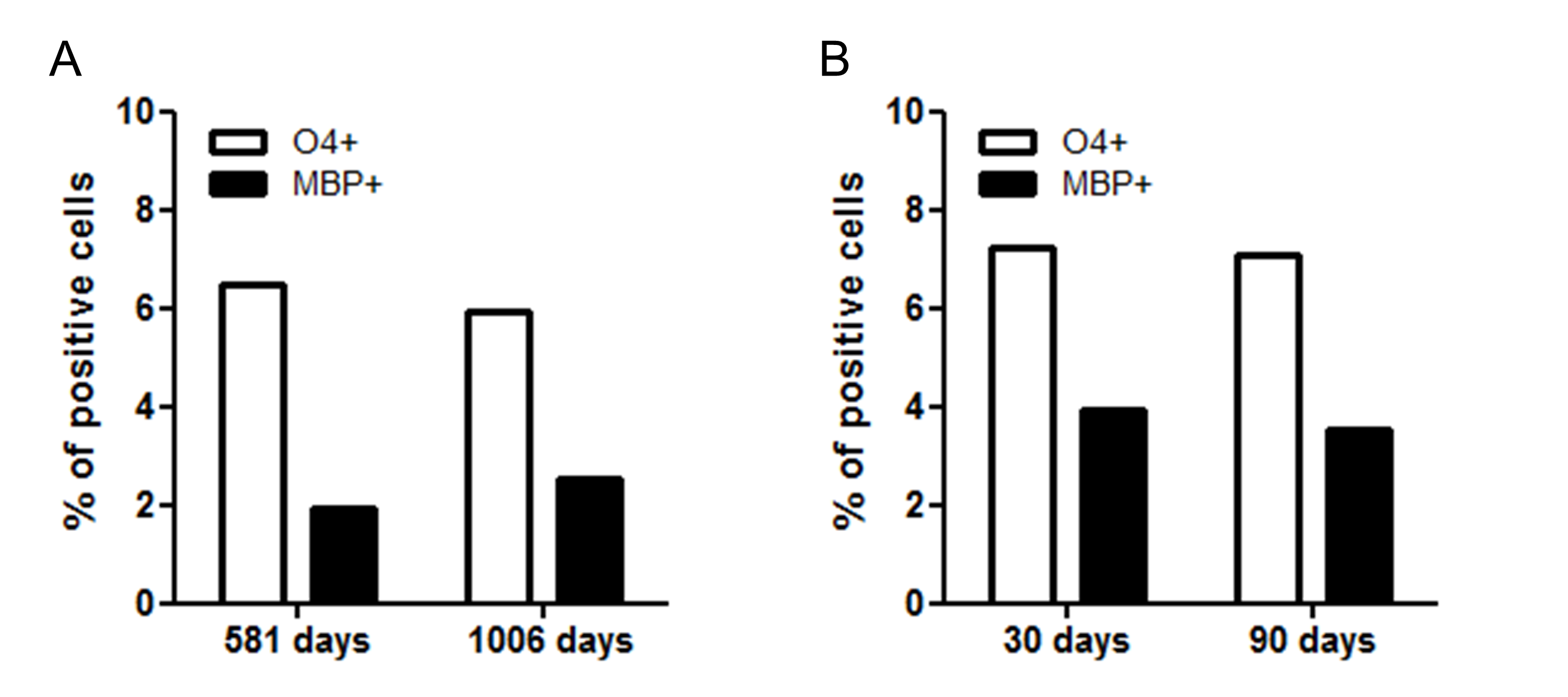

Supplement: S6 Fig — (A) CMK6SFF-derived OPCs cryopreserved for both 581 days and 1006 days were differentiated into oligodendrocytes. (B) A3-derived OPCs cryopreserved for both 30 days and 90 days were differentiated into oligodendrocytes. O4- and MBP-positive cells (O4+, MBP+) were counted and normalized by nuclei number. (TIF) [file pone.0171947.s006.tif]

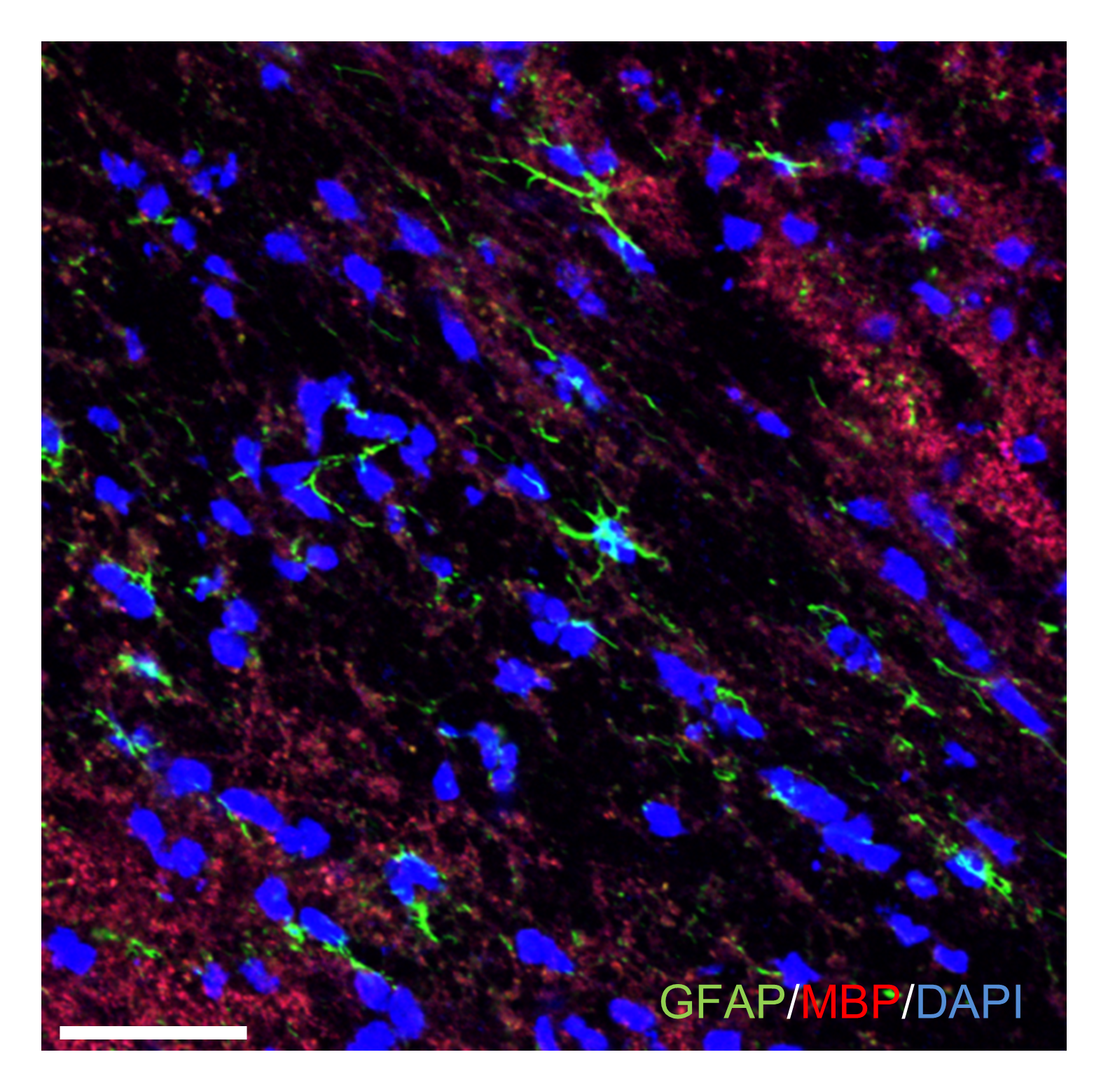

Supplement: S7 Fig — Distribution of CMK6SFF-derived cells transplanted into the corpus callosum of the cerebral cortex in neonatal mice. Sections stained with GFAP (green), MBP (red), and DAPI (blue). Scale bars = 50 μm. (TIF) [file pone.0171947.s007.tif]
